# Current Understanding of the Interplay between Phytohormones and Photosynthesis under Environmental Stress

**DOI:** 10.3390/ijms160819055

**Published:** 2015-08-13

**Authors:** Mayank Anand Gururani, Tapan Kumar Mohanta, Hanhong Bae

**Affiliations:** School of Biotechnology, Yeungnam University, Gyeongsan, Gyeongbook 712-749, Korea; E-Mail: nostoc.tapan@gmail.com

**Keywords:** environmental stress, phytohormones, photosystem II (PSII) repairing system, photosystem II, stress tolerance

## Abstract

Abiotic stress accounts for huge crop losses every year across the globe. In plants, the photosynthetic machinery gets severely damaged at various levels due to adverse environmental conditions. Moreover, the reactive oxygen species (ROS) generated as a result of stress further promote the photosynthetic damage by inhibiting the repair system of photosystem II. Earlier studies have suggested that phytohormones are not only required for plant growth and development, but they also play a pivotal role in regulating plants’ responses to different abiotic stress conditions. Although, phytohormones have been studied in great detail in the past, their influence on the photosynthetic machinery under abiotic stress has not been studied. One of the major factors that limits researchers fromelucidating the precise roles of phytohormones is the highly complex nature of hormonal crosstalk in plants. Another factor that needs to be elucidated is the method used for assessing photosynthetic damage in plants that are subjected to abiotic stress. Here, we review the current understanding on the role of phytohormones in the photosynthetic machinery under various abiotic stress conditions and discuss the potential areas for further research.

## 1. Introduction

Plants as sessile organisms are often subjected to various environmental stresses that sometimes lead to enormous crop losses [1]. Crop losses have increased manifold over the past decades due to soil and atmospheric stress factors such as high/low salinity, drought, freezing, high temperatures, and heavy metal toxicity [2]. However, in order to overcome such adverse effects, plants have evolved elaborate mechanisms of acclimation and adaptation that confer varying degrees of stress tolerance to them. Moreover, with the advent of various plant genetic engineering techniques, it is now possible to design stress-tolerant plants with a modified plant architecture and metabolism. However, to achieve this, it is important to have a comprehensive understanding of how plants respond to specific stresses at their physiological and molecular levels. At present, with the extensive scientific literature available, it is well-known that abiotic stress induces changes in photosynthesis and phytohormone signaling and that these changes are often interlinked [3,4,5].

Photosynthesis is one of the most important processes that gets severely affected with the onset of abiotic stress. Abiotic stress results in the over-reduction of the electron transport chain (ETC) which, in turn, leads to photo-oxidation [6,7]. In order to survive under these conditions, plants go through a mechanism where excessively absorbed light energy is converted into thermal energy and an optimal rate of electron transport is achieved. The process of the dissipation of excess energy in the form of heat is commonly referred to as non-photochemical quenching (NPQ) [8,9,10,11,12,13,14,15]. The photosystem II (PSII) protein complex is the most vulnerable component of the photosynthetic machinery to abiotic stress. Photodamage to the PSII complex is an unavoidable process in all the photosynthetic organisms; however, these organisms have developed a process to prevent the over-accumulation of damaged PSII complex [16,17] by recovering the damaged PSII via the PSII repairing system. Therefore, at present, the PSII damage repair mechanism has gained a lot of attention among researchers. Also, there is evidence suggesting that various molecules of reactive oxygen species (ROS), which are generated in the chloroplast as a consequence of abiotic stress, do not damage the PSII complex directly but hamper the synthesis of the D1 protein of PSII complex after the stress-induced photoinhibition [18,19,20].The D1 protein is one of the two reaction center proteins of PSII that is involved in PSII repair during photoinhibition [8]. The D1 protein binds cooperatively to the other reaction center protein, D2, and carries cofactors essential for electron transport from the oxygen-evolving complex (OEC) to the plastoquinone pool [21]. The proteolysis synthesis of new copies of D1 protein is essential for the survival of plants under abiotic stress-induced photoinhibition (reviewed in [8,20]).As mentioned earlier, photosynthesis is the most vulnerable process that gets affected by any adverse environmental condition; hence, it is reasonable to measure photosynthetic parameters in order to study the response of plants and the effect of hormone application during stress conditions. Chlorophyll-a transient kinetic analysis through the so-called OJIP (where O and P denote origin and peak, respectively, and J and I are the intermediate phases of fluorescence induction) test is one of the most common methods that is being used by researchers to assess plant performance under conditions of stress [22,23,24,25,26]. It is a non-invasive analysis tool commonly used for analyzing the effects of a range of stress factors on plants and provides relevant details about PSII photochemistry and the events in ETC. A series of mathematical calculations is then used to simplify this information via specialized software. Because it is a simple method and gives detailed information on plants’ photosynthetic components under various stress conditions, OJIP analysis has gained wide acceptance over the past few years [15,26,27,28,29,30,31,32].

Recent studies have shown that a complex interplay between phytohormones and cellular redox machinery regulates the response of the photosynthetic units to different abiotic stress conditions [33,34,35,36,37]. Investigations on *Arabidopsis* mutants with defective light-harvesting complex (LHC) under high irradiation revealed a strong interaction between the control of transfer of excitation energy and the hormonal regulation [38]. Furthermore, the expression of plastidial and nuclear genes involved in photosynthesis can be under hormonal regulation [4]. Recent findings suggest a direct involvement of cytokinins (CKs) in the PSII damage repair process [39]; hence, it is imperative to consider the changes in photosynthesis and levels of hormones in plants subjected to various environmental stress conditions as an interlinked process and not as isolated events. However, the highly complex molecular linkages between the signaling networks of various hormones make it difficult to elucidate the precise roles of individual hormones in regulating the expression of photosynthetic genes and in the regulation of the PSII damage repair process. This review summarizes and evaluates the current understanding of the influence of different phytohormones on the photosynthetic machinery under environmental stress. It also highlights the potential areas for further research in the involvement of hormonal regulatory networks of photosynthetic machinery in response to abiotic stress.

**Table 1:** Recent studies on phytohormones reported to affect various components of photosynthetic machinery in different plants under abiotic stress conditions.

Hormone	Plant	Stress	Effect on Photosynthetic Components	Reference
ABA	*Arabidopsis*	High light	Reduced expression of photosynthetic genes	[40]
	*Arabidopsis*	Chemical (norflurazon)	Induction of genes encoding LHCB proteins	[41]
	*Arabidopsis*	Drought	Positive regulation of genes encoding LHCB proteins	[42]
	Barley	Heat	Decreased heat damage of chloroplast ultrastructure, improved PSII efficiency	[43]
	Barley	Low temperature	Higher photochemical quenching and NPQ	[44]
	Common bean, tobacco, beetroot, maize	Drought	Improved PSII efficiency	[45]
	Rice	Drought	Improved NPQ and PSII efficiency	[46]
	Rice, cabbage	High salinity	Enhanced PSII efficiency, NPQ and PSII photochemistry	[47]
	*Lycium chinese*	Drought	Slower decline in PSII efficiency, improved NPQ	[48]
Auxin	*Arabidopsis*	Drought	Improved maximal electron transfer rate, photochemical quenching and maximal photochemical yield of PSII	[49]
	Sunflower	Heavy metal	Increased ability of energy trapping by PSII reaction centres	[50]
BRs	Rice	High salinity	Prevention of photosynthetic pigment loss	[51]
	Mustard	Heavy metal	Higher chlorophyll accumulation and improved *P*_N_	[52]
	Winter rape	Heavy metal	Improved energy absorption, trapping, and electron transport by PSII reaction centers. Efficient oxygen-evolution	[53]
BRs	Mungbean	Heavy metal	Higher *P*_N_ and improved stomatal conductivity	[54]
	Cucumber	Drought	Higher PSII efficiency, improved NPQ	[55]
	Tomato	Chemical stress, heavy metal	Improved *P*_N_, PSII efficiency, and NPQ	[56]
	Tomato	Heat	Improved recovery of *P_N_*, stomatal conductance, and maximum carboxylation rate of Rubisco, electron transport rate, relative quantum efficiency of PSII photochemistry, photochemical quenching, and increased NPQ	[57]
	Pepper	Drought	Improved utilization and dissipation of excitation energy in the PSII antennae. Alleviation of drought-induced photoinhibition	[58]
CKs	Tobacco	Drought	Slower degradation of photosynthetic protein complexes, increased expression of genes associated with PSII, Cytb6f complex, PSI, NADH oxidoreductase, and ATP synthase complex	[59]
	*Arabidopsis*	High light	Reduced PSII efficiency, low accumulation of D1 protein	[39]
	Maize	Drought	Increased electron donation capacity of PSII, higher plant photosynthetic performance index, energy absorption and trapped excitation energy	[60]
ET	Mustard	Heavy metal	Efficient PSII, *P*_N_, stomatal conductance, and Rubisco activities	[61]
	Mustard	Low nitrogen	Improved *P*_N_, Rubisco activity, and stomatal conductivity	[62]
	Mustard	High salinity	Higher photosynthetic-nitrogen and sulfur use efficiency and improved quantum yield efficiency of PSII	[63]
	Mustard	Heavy metal	Increased maximal quantum efficiency of PSII, *P*_N,_and Rubisco activity	[64]
	Tobacco	High salinity, oxidative stress	Increased *P*_N_	[65]
GAs	Wheat	High salinity	Improved *P*_N_ and stomatal conductance	[66]
	Mustard	High salinity	Increased photosynthetic efficiency and stomatal conductance	[67]
	Linseed	High salinity	Improved *P*_N_, and stomatal conductance	[68]
	Sunflower	Heavy metal	Increased ability of energy trapping by PSII reaction centers	[50]
JA	Rice	High salinity	Improved leaf water potential, *Fv/Fm*, and *P*_N_	[69]
	*Pisum sativum*	High salinity	Increased non-variable fluorescence, *Fv/Fm*, and Rubisco activity	[70]
	Barley	High salinity	Improved *Fv/Fm* and *P*_N_	[71]
	*Arabidopsis*	Heavy metal	Improved photosynthesis activity	[72]
	*Arabidopsis*	Heavy metal	Improved PSII activity, *Fv/Fm*, and *P*_N_	[73]
SA	*Arabidopsis*	Drought	Higher *P*_N_, maximum efficiency of PSII, and maximum quantum yield of PSII	[74]
	Wheat	High salinity	Increased quantum yield of PSII	[75]
SA	Wheat	Heat, high light	Improved PSII efficiency, slower degradation and accelerated recovery of damaged D1 protein	[76]
	Wheat	Drought	Upregulated expression of luminal, oxygen-evolving enhancer, and PSII assembly factor proteins	[77]
	Rice	Drought	Higher *P*_N_, stomatal conductance, and transpiration rate	[78]
	Mustard	High salinity	Improved *P*_N_, stomatal conductance, and water use efficiency	[79]
	Mustard	High salinity	Improved PSII efficiency,*P*_N_, Rubisco activity, water-use efficiency, and stomatal conductance	[80]
	Cotton	High salinity	Increased PSII activity, *P*_N_ and transpiration rate	[81]
	Maize	High salinity	Increased *P*_N_ and Rubisco activity	[82]
SA	Grapevine	Heat	Improved *P*_N_, chlorophyll *a* fluorescence, higher stomatal conductance	[83]
	Grapevine	Heat	Improved *P*_N_, enhanced Rubisco and PSII activities	[84]
	Tomato	Drought	Higher *P*_N_, stomatal conductance	[85]
	Common sage	Drought	Maintenance of maximum efficiency of PSII and protection of photosynthetic apparatus	[86]
	*Torreyagrandis*	High salinity	Increased *P*_N_	[87]
SLs	*Arabidopsis*	Drought	Higher expression of photosynthetic genes	[88]

ABA, abscisic acid; ATP, adenosine triphosphate; BRs, brassinosteroids; CKs, cytokinins; Cytb6f, cytochrome b6f complex; D1, PSII protein encoded by *PsbA* gene; ETC, electron transport chain; GAs, gibberellic acids; HL, high light; JA, jasmonic acid; LHCB, light-harvesting chlorophyll a/b binding protein; NADH, nicotinamide adenine dinucleotide + hydrogen (reduced); NPQ, non-photochemical quenching; *P*_N_, net photosynthesis rate; PSI, photosystem I; PSII, photosystem II; Rubisco, ribulose-1,5-bisphosphate carboxylase oxygenase; SA, salicylic acid; SLs, strigolactones. *Fv*, variable fluorescence; *Fm*, maximum fluorescence.

## 2. PSII Damage and Role of Hormones

PSII is the most vulnerable component of the photosynthetic machinery to abiotic stress [8,89,90,91]. However, plants have evolved an efficient PSII repairing system that facilitates their survival under adverse environmental conditions. PSII damage repair is a complex cyclic process that involves several protein kinases, phosphatases, and proteases that facilitate the phosphorylation and subsequent dephosphorylation of D1 protein of the PSII complex [92,93,94]. Although earlier studies on the PSII damage repairing system have revealed the precise mechanism and the factors involved to a large extent [7,13,18,21,94,95], much of it remains to be investigated, particularly in terms of direct involvement of hormonal signaling. Nevertheless, several recent studies have indicated that different hormones (Table 1) and the transcription factors regulated by these hormones (reviewed in [20]) can modulate the expression of genes involved in photosynthesis, the efficiency of the PSII complex, and the accumulation of chlorophyll under abiotic stress [35,59,96,97,98,99]. However, it is critical to note that the intricate nature of crosstalk between various hormonal pathways makes it extremely difficult to elucidate the key roles of specific hormones in fine-tuning the expression of photosynthetic genes and the regulation of the PSII damage repair system. For example, glycine betaine (GB), a molecule belonging to a group of compatible plant osmolytes, is known to protect the photosynthetic apparatus under stress conditions primarily by stabilizing the PSII complex and preventing the degradation of lipids and enzymes required in maintaining a balance in ETC [100,101,102,103]. Although GB is not directly involved in plants’ growth and developmental processes, experimental evidence suggests that altered accumulation of GB can influence the endogenous production of several stress-responsive plant hormones [104,105]. GB is known to protect various proteins, lipids, and enzymes critical for the photosynthetic machinery. Biosynthesis of GB is associated with the endogenous levels of abscisic acid (ABA), salicylic acid (SA), and ethylene (ET). Accumulation of GB has been associated with the signaling network of major phytohormones such as ABA, ET, and SA under various abiotic stress conditions [84,106,107,108]. Similarly, several other recent reports have indicated that many phytohormones play a significant role, either directly or indirectly, in regulating the photosynthetic machinery under abiotic stress [20] (Table 1).Crosstalk between these hormones was well studied for their roles in plant development and stress tolerance; however, the interaction between these hormones with GB is not clearly understood.

### 2.1. Abscisic Acid

**Figure 1:**
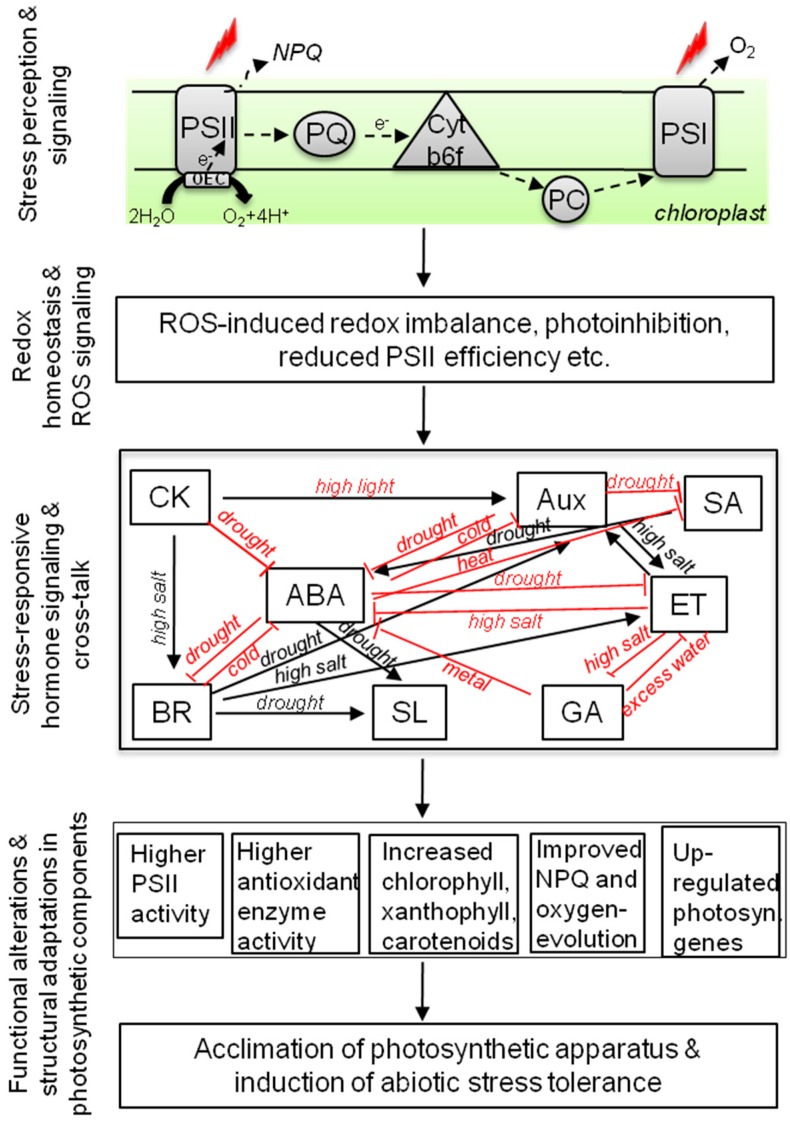
Involvement of stress-responsive phytohormones and their crosstalk in the protection of photosynthetic machinery under abiotic stress conditions. Abiotic stress factors (indicated as red lightning symbols) mainly target the photosynthetic reaction centers, photosystem II (PSII), and photosystem I (PSI). PSII is the most susceptible reaction center as it is involved in the water-splitting process of photosynthesis where water is converted to oxygen and protons through the oxygen-evolving complex (OEC) and the electrons (e^−^) are released and transferred to the PSI complex via plastoquinone (PQ), cytochrome b6f complex (Cytb6f), plastocyanin (PC), and several other intermediatory molecules. Under stressful conditions, plants dissipate the excess energy in the form of heat through the light-harvesting antenna molecules and this mechanism is known as non-photochemical quenching (NPQ). Abiotic stress-induced photoinhibition creates a redox imbalance and reactive oxygen species (ROS) molecules are generated that are highly detrimental for the plant cells. In addition, ROS molecules inhibit the PSII damage-repair process. With the onset of abiotic stress in plants, the phytohormone signaling also gets altered and the endogenous levels of these hormones change according to the intensity and duration of specific stress. A highly complex crosstalk between these hormones exists where some of these hormones have up-regulatory interaction (indicated as black arrows) with each other while others have inhibitory relations (indicated as blunt arrows in color). Hormonal crosstalks in plant cells under stressful conditions are known to affect protective mechanisms (e.g., antioxidative machinery) that facilitate the survival of plants under stress. In addition, these hormonal crosstalks and individual hormonal activities regulate the photosynthesis under stress conditions by inducing changes to various photosynthetic components. ABA: abscisic acid; CK: cytokinin; BR: brassinosteroid; SL: strigolactone; SA: salicylic acid; GA: gibberellic acid; ET: Ethylene; Aux: auxins.

ABA is a well-known phytohormone that not only plays a significant role in plant’ developmental processes such as seed dormancy, embryo maturation, stomatal closure, and senescence but also in promoting tolerance against diseases and abiotic stress conditions [109,110,111,112]. In addition, ABA is known to regulate photosynthesis and mediate the mobilization of the photosynthate pool between sources and sink tissues [113,114]. Moreover, pretreatment of seedlings with ABA has been reported to increase chlorophyll and carotenoid accumulation, and it also maintains optimal efficiency of the PSII complex in plants subjected to water stress [45]. Photosynthetic oxygen evolution regulated by the OEC of the PSII complex is the most pivotal process required for the survival of life on earth [115,116]. The *in vivo* effect of ABA on photosynthetic oxygen evolution in barley leaves revealed that ABA influences the functioning of PSII reaction centers by disrupting the granal chloroplast [3]. Partial protection of the PSII photochemistry against photoinhibition at low temperatures was reported in ABA-treated barley seedlings [44]. Similarly, exogenous application of ABA in barley leaves under heat stress showed a significant reduction in the heat damage to the chloroplast structure. The study of fluorescence parameters further revealed that the heat-induced increase in the initial fluorescence (*F*o) was virtually eliminated in ABA-treated leaves, thus indicating a direct involvement of ABA in maintaining the thermostability of the PSII complex under heat stress [43]. It must be noted here that xanthophylls present in light harvesting complex II (LHCII) play critical roles in the protection of photosynthetic organelles against excess light by facilitating efficient quenching of excited chlorophyll molecules [117,118]. Interestingly, violaxanthin, which acts as a common precursor of ABA biosynthesis, has also been reported to be involved in the protection of photosynthetic machinery under high salinity and high-light stresses (reviewed in [119]). Exogenously-fed ABA has been reported to alleviate the salt stress-induced decline in the efficiency of PSII photochemistry. In addition, a lower reduction state of the PSII complex, an enhanced capacity of NPQ, and an increased xanthophyll cycle pool size was observed [47]. This was in agreement with a previous study that demonstrated the role of ABA in protecting the xanthophyll cycle pool and the photosynthetic apparatus from photo-oxidative stress [46]. More recently, a novel *Lycium chinense*-derived violaxanthin de-epoxidase (*LcVDE*) gene was characterized and reported to have a close correlation with drought-induced endogenous ABA accumulation [48]. The relative expression of *LcVDE* and the de-epoxidation rate of xanthophyll carotenoids and NPQ were found higher under drought conditions. However, these elevations were reduced by the application of a potent ABA inhibitor [48]. Moreover, transgenic *Arabidopsis* lines expressing *LcVDE* showed improved drought tolerance and a slower decrease in the maximum quantum yield of primary photochemistry of PSII (*Fv/Fm*) compared to control plants [48]. Because violaxanthin de-epoxidase (VDE) plays an important role in the conversion of violaxanthin to zeaxanthin and violaxanthin serves as a precursor for ABA biosynthesis, it can be speculated that ABA could regulate the expression of photosynthetic genes under drought stress (Figure 1).

Studies have demonstrated that beside harvesting light, proteins in LHC also play a photoprotective role in plants by maintaining equilibrium between the excitation energies of PSI and PSII [120,121,122]. Xu *et al.* [42] demonstrated a close interrelationship between the expression of LHC proteins and ABA signaling (Figure 1). The inhibition of light-harvesting chlorophyll a/b binding protein (LHCB) protein expression was attributed to the reduced responsiveness of stomatal movement to ABA and the reduced drought tolerance in *Arabidopsis* plants, possibly due to altered ROS homeostasis [42]. Several previous studies conducted on various plants at different developmental stages unanimously point out that ABA treatment downregulates the expression of several genes of the LHCB protein family [40,123,124,125]. In contrast, another study showed that the application of ABA at low concentrations can enhance the expression of *LHCB* [41]. A possible reason for the inconsistency between these studies could be the choice of different plant species, different plant developmental stages, or some additional experimental factors influencing LHCB expression.

PSII complex has often been referred to as “the engine of life on earth” [8] mostly because of its role in photosynthetic oxygen evolution. So far, 25 genes have been identified that encode the PSII core protein complex, and out of them, very few have been studied extensively in plants and other photosynthetic organisms [126,127]. The 33 kDa protein photosystem b (PsbO), which is one of the main proteins of OEC, is known to influence plant growth and other developmental processes in plants [9,14,128,129]. Transgenic potato plants with downregulated expression of *PsbO* showed improved abiotic stress tolerance under high salinity, heavy metal toxicity, and drought stresses. The over-accumulation of ABA in transgenic potato lines under the aforementioned stress conditions indicates a correlation between ABA signaling and *PsbO* expression [14]. A similar relationship between the *PsbO* gene and the acclimation of the photosynthetic apparatus under abiotic stress conditions in the forage grasses *Festuca arundinacea* and *F. pratensis* has been documented [130]. The application of cyprosulfamide, a synthetic growth-promoting regulator, either alone or in combination with ABA, is reported to induce salt tolerance in rice plants. Interestingly, the combination of ABA and cyprosulfamide prolonged the expression of stress-responsive genes beyond the stress period, and facilitated the plants in maintaining their improved growth. Proteomic analyses revealed that several photosynthetic proteins, including photosystem b *P* protein (PsbP) and PsbO, were either repressed or induced with the exogenous application of ABA in combination with cyprosulfamide, further indicating a strong interaction between ABA signaling and the regulation of the photosynthetic apparatus under environmental stress [131]. It is apparent with the findings discussed above that ABA signal perception and transduction are closely associated with redox signaling and other signaling pathways. Future efforts toward understanding these molecular interactions can greatly facilitate the development of stress-tolerant crops. 

### 2.2. Auxins

Auxins are a group of plant hormones with many vital roles in plant growth and developmental processes. Auxins, such as indole butyric acid (IBA), have been demonstrated to regulate plants’ responses to drought, its photosynthetic apparatus, and the chloroplast structure [132,133]. Transgenic *Arabidopsis* plants ectopically expressing *UGT74E2* encoding IBA-UDP-glucosyltransferase displayed an enhanced accumulation of IBA and IBA-glucosyltransferase under osmotic stress [49]. Transgenic plants showed an increased maximal electron transport rate and photochemical quenching under dehydration conditions, thus indicating the influence of auxin signaling on photosynthesis and on the morphology of the plants. The tomato *sulfurea* mutant plants, which suffer from partial auxin auxotrophy and reduced chlorophyll, showed a PSII quantum yield similar to the control plants; the amounts of reaction center chlorophyll for PSI (P700) and the ratio of plastocyanin (PC) to P700 (PC/P700) were notably lower than the control plants, indicating that PSI levels in these mutant plants were significantly reduced [134].

Pre-treatment of wheat seeds with indole acetic acid (IAA, an auxin) has been reported to ameliorate the effects of cadmium (Cd)-induced heavy metal toxicity via enhanced activities of ROS-scavenging enzymes [135]. Although the authors did not analyze the photosynthetic activities, improved growth parameters were proposed to be associated with the increased accumulation of photosynthetic pigments such as chlorophyll and carotenoids in IAA-treated plants [135]. In a similar study, IAA-treated maize seedlings showed upregulated activities of ROS-scavenging enzymes and improved plant growth under lead-induced heavy metal stress [136]. Interestingly, the increased accumulation of carotenoids and other low molecular weight metabolites in plants has been proposed to inhibit the generation of singlet oxygen [9,137]. Since the generation of ROS was inhibited in IAA-treated plants, it likely facilitated the *de novo* synthesis of D1 protein during the PSII repair cycle, resulting in the improved growth of maize seedlings. IAA-treated sunflower plants subjected to excessive copper (Cu) stress showed a decrease in growth inhibition primarily because of the net photosynthetic rates (*P*_N_) and the quantum yield of dark-adapted PSII (*Fv*/*Fm*) [50]. Despite a significant number of studies on auxin signaling under abiotic stress, the relationship between auxin biosynthesis and its influence on the photosynthetic machinery under environmental stress remains unclear and warrants further research.

### 2.3. Brassinosteroids

Brassinosteroids (BRs) are plant hormones that are known for a wide range of functions in plant defense, growth and development, abiotic stress tolerance, and photosynthetic carbon fixation [58,138,139,140,141,142]. Several studies on BR-mutant cell lines have suggested that not only do BRs play a significant role in plant growth and development, but they also regulate the architecture of the photosynthetic apparatus, photosynthetic oxygen evolution, and PSII quantum yield under normal and stress conditions [34,35,143,144] (Figure 1). Yu *et al.* [55] examined the effect of BR application on photosynthesis in *Cucumissativus* and reported a marked increase in the quantum yield of PSII electron transport, higher *Fv*/*Fm*, and increased activity of ribulose-1,5-bisphosphate carboxylase/oxygenase (Rubisco) [55]. They found that the photosynthetic activity of *C. sativus* plants was inhibited with the treatment of brassinazole, an inhibitor of the biosynthesis of BR. In another study, BR was found to upregulate and brassinazole was found to downregulate the expression of photosynthetic genes, including those encoding for the Rubisco enzyme [145]. These findings indicate that the growth-promoting activity of BRs can be partly corroborated with the improved photosynthesis. The characterization of *Arabidopsis* mutants expressing a brassinosteoid insensitive 1 gene (*BRI1*(*Y831F*)) revealed that in addition to increased leaf size, they also showed higher *P*_N_ [143]. Similarly, the significantly reduced expression of photosynthetic genes coupled with reduced plant growth and lower photosynthetic activity was observed in *Arabidopsis BRI1* mutants [146]. Reduced photosynthetic efficiency, disrupted PSII assembly, low photosynthetic oxygen evolution and PSII quantum yield, and enlarged thylakoids were also reported in *Arabidopsis BR1OE* and *BR1-116* mutants, further indicating the role of BRs in the regulation of photosynthesis in higher plants [34].

Beside studies on mutant plants, the exogenous application of BRs has been studied extensively in various crops under normal and abiotic stress conditions (Table 1). BRs alleviated Cd-induced metal toxicity on the primary photochemistry of PSII in rape cotyledons by maintaining efficient ETC and limiting the damage in OEC and PSII reaction centers [53]. Several other reports are documented where BR was shown to mitigate the loss of chlorophyll molecules and the reduction in carbonic anhydrase activity in various crops exposed to Cd- or aluminum-induced metal toxicity [51,52,54,147].

The exogenous application of BRs has also been reported to induce thermotolerance in various plants. For example, BR-treated tomato plants exposed to high-temperature stress showed higher activity of ROS-scavenging enzymes [57]. The analysis of photosynthetic parameters revealed that BR treatment alleviated heat-induced inhibition of photosynthesis relatively better than the control plants. Furthermore, the reduced NPQ and improved *P*_N_, stomatal conductance, Rubisco carboxylation, photochemical quenching, and relative quantum efficiency of PSII was recorded in BR-treated tomato plants [57]. Another study on *Cucumis sativus* plants showed improved utilization of absorbed light energy in chloroplasts and reduced drought-induced photoinhibition [145]. This could be attributed to an efficient dissipation of excitation energy in the form of heat from the LHCII antenna as evidenced with the reduced NPQ in BR-treated *C. sativus* plants [145]. Given these findings, it becomes interesting to understand the effect of BR application on various components of the photosynthetic apparatus. Although it is well documented that BR application indeed improves the photosynthetic efficiency and that BRs regulate the photosynthesis under normal and abiotic stress conditions (reviewed in [148]), the precise mechanism underlying BR-induced effects on photosynthetic components remains elusive. Rothová *et al.* [149] investigated the effects of BR application on various parts of photosynthetic events in maize and spinach. Interestingly, the results revealed that although the efficiency of the photosynthetic ETC responded negatively to BR treatment in both plants, responses regarding PSII activity were entirely different. While a positive effect of BR treatment was noted in the OEC and PSII units in maize, contradictory results were reported in spinach. Similarly, the maize plants exhibited a positive influence of BR treatment on the accumulation of photosynthetic pigments; however, this was not true for the spinach plants [149]. These findings pose an important question regarding the possible differences in the PSII response to BR treatment in various plants. Another question could be whether hormonal crosstalk existing in certain plant species might not occur in other species. More studies on the effect of BR treatment on individual components of plant photosynthetic machinery could resolve the relationship between hormone signaling and its influence on PSII.

### 2.4. Cytokinins

Cytokinins (CKs) have been described as a group of phytohormones primarily responsible for the cell division in roots and shoots. Given that several CK biosynthetic pathways are located in the plastids, they play a crucial role in chloroplast biogenesis [150]. Previous studies have indicated a role for CKs in conferring abiotic stress tolerance in higher plants, presumably via modulating the regulatory mechanism of photosynthetic processes [59,151,152] (Figure 1; Table 1). The OJIP analysis of CK-treated maize plants exposed to drought conditions revealed that the electron-donating capacity of PSII and the photosynthetic performance index was increased while electron transport to the acceptor side of PSII was reduced with the application of CK [60]. Transgenic tobacco lines over-accumulating CKs exposed to severe drought conditions displayed increased expression of photosynthetic genes encoding proteins of PSI, PSII, and cytochrome b6f (Cytb6f) complex, indicating that endogenous CKs might protect the photosynthetic machinery under stressful conditions [59,152] (Figure 1). Recently, Cortleven *et al.* [39] demonstrated that *Arabidopsis* mutants with decreased production of endogenous CKs were more susceptible to high-light stress as the levels of D1 protein were markedly decreased in mutant plants. Furthermore, the efficiency of enzymatic and non-enzymatic scavenging systems that regulate the photoprotective mechanisms was also significantly reduced in the mutant *Arabidopsis* plants [39]. This evidence clearly illustrates that CKs play a pivotal role in regulating the photoprotective mechanism under high-light stress in higher plants. Incidentally, the altered accumulation of endogenous CK levels appears to have little or no influence on the structural composition of the subunits of the supercomplex as revealed by the proteomic analyses of transgenic tobacco plants with increased and decreased accumulations of endogenous CKs [5]. Nonetheless, it would be interesting to determine whether CKs also play the same role in a plant’s response to photoinhibition that is induced by abiotic stress factors.

### 2.5. Ethylene (ET)

Ethylene (ET) is a gaseous phytohormone that is involved in several physiological processes, including photosynthesis, plant growth, biotic and abiotic stress responses, and senescence [61,153,154] (Figure 1). However, the over-accumulation of ET under adverse environmental conditions is known to cause oxidative stress in plant cells that in turn inhibits photosynthesis [153]. An earlier study has also suggested a role of ET in protection against, or repair of, heat-induced photo-oxidative stress in *Arabidopsis* [155]. Furthermore, ET has been shown to act as an amplifier for ROS accumulation, indicating a synergistic effect between ROS production and ET biosynthesis in plants [65] (Figure 1). Transgenic tobacco lines producing reduced levels of endogenous ET exhibited less accumulation of ROS and improved *P*_N_ [65]. Furthermore, the exogenous application of ethephon, an ethylene-releasing compound, significantly influenced photosynthesis, stomatal conductance, and growth of mustard cultivars under nitrogen-deficient and normal conditions through improved nitrogen use efficiency [62,156]. Similar results were reported under high-nitrogen levels, and it was suggested that the application of ethephon influences growth, photosynthesis, and nitrogen accumulation in plants [157]. The increased accumulation of ET under nitrogen-deficient conditions was attributed to reduced photosynthesis and nitrogen use efficiency. Moreover, the involvement of ET in the alleviation of the salt stress-induced reduction in photosynthesis by the application of sulfur has also been reported in mustard plants [65]. The study showed that ET regulates plants’ responses and excess sulfur promotes photosynthesis and induces salt-stress tolerance [63]. Similarly, higher *Fv*/*Fm* and improved *P*_N_ were reported in mustard plants under Cd-induced heavy metal stress that was associated with sulfur-induced activation of the antioxidant system through ET signaling [64]. To date, there is no consensus among researchers in terms of altered ET signaling in plants under different stress conditions. Therefore, in addition to investigating the photoprotective role of ET, it would be excellent to elucidate the cross point of interaction between other major phytohormones and ethylene signaling pathways.

### 2.6. Gibberellins

Gibberellic acids (GAs) or gibberellins are a group of plant growth hormones generally involved in the induction of photosynthesis, abiotic stress tolerance, and developmental processes such as seed germination, fruit development, leaf expansion, stem elongation, and flowering [158,159,160]. Transgenic *Arabidopsis* plants with an over-accumulation of endogenous GAs have been reported to exhibit longer hypocotyledonous stems, increased internodal length, pale green leaves, and early flowering [161]. Earlier studies on the regulatory role of GAs on photosynthetic parameters under abiotic stress presented a contradicting picture where some researchers demonstrated a significant effect of GA on photosynthesis, while others reported no such effect. For instance, Ashraf *et al.* [66] reported improved photosynthetic capacity, alleviation of the adverse effects of high-salt stress, ion accumulation and vegetative growth, and increased biomass in GA_3_-treated wheat cultivars. Similarly, the short-term application of GA_3_ in soybean and broad bean plants resulted in increased *P*_N_, increased stomatal conductance, improved photosynthetic oxygen evolution, and increased carboxylation efficiency [160]. Improved photosynthesis in these plants was attributed to the increased activity and content of ribulose-1,5-bisphosphate carboxylase (RuBPCase, an enzyme that regulates photosynthetic carbon fixation) which stimulated the synthesis of Rubisco subunits. In contrast, Dijkstra *et al.* [162] investigated the relationship between relative growth rate and endogenous GAs in two inbred lines of *Plantago major* plants producing enhanced and reduced levels of GAs, and reported that although GA treatment promoted the vegetative growth, the chlorophyll a content and photosynthetic activity per unit leaf area were reduced, indicating the possible involvement of some other regulatory factors [162]. Similarly, no correlation was found between *P*_N_ and endogenous GAs in GA-deficient and control tomato plants [163]. Another study in transgenic tobacco lines with increased and reduced levels of bioactive GAs suggested a positive correlation between photosynthetic activity and endogenous GA levels in higher plants [164]. The inconsistency regarding the regulatory role of GAs in photosynthesis could be attributed to the different biosynthetic steps that were affected in the mutant lines as those modifications may or may not affect active GAs; they may also affect the GAs that specifically regulate certain processes and not others.

Interestingly, studies on the influence of GAs on photosynthesis under abiotic stress strongly indicate that GAs do play a crucial role in altering the photosynthetic efficiency of plants under stress. The foliar application of GA_3_ was reported to counteract the adverse effects of high salinity with the accumulation of chlorophyll, proline, and the improved activity of ROS-scavenging enzymes in maize [165]. The application of GA_3_ increased the photosynthetic efficiency, dry mass, leaf chlorophyll content, and stomatal conductance in mustard plants exposed to salt stress [67]. The exogenous application of GA_3_ was reported to mitigate the detrimental effects of salt stress and displayed improved *P*_N_, higher chlorophyll content, and increased biomass in mustard plants [166]. The application of GA_3_ in combination with calcium chloride also resulted in reduced membrane damage and lipid peroxidation, improved photosynthesis, increased chlorophyll accumulation, and an efficient antioxidant system in linseed plants subjected to salinity stress [68]. In another report, improved *P*_N_, transpiration rate, stomatal conductance, and water-use efficiency were noted in GA_3_-treated seeds of two wheat cultivars exposed to salt stress [159].

Several reports have documented the improved stress tolerance in plants exposed to heavy metal toxicity through increased photosynthetic activity with the application of Gas [50,167,168]. A detailed analysis of major photosynthetic parameters in sunflower plants exposed to high-Cu stress revealed that GA treatment increased energy trapping by PSII reaction centers and also increased the stability of the LHCII complex as well as *P*_N_, and *Fv*/*Fm* were significantly higher, indicating the photoprotective role of Gas [50]. The addition of GA_3_ to a nutrient solution containing Cd that was used for growing *Glycine max* plants alleviated the growth-inhibitory effects of Cd stress, which was evident with improved plant growth rate, net CO_2_ assimilation rate, and increased chlorophyll content, indicating the role of GAs in improved photosynthesis [167]. It must be noted that Cd toxicity is believed to be associated with altered stomatal density, photosynthetic oxygen evolution, and ultrastructural changes in thylakoids [169,170]. Hence, it would be interesting and worth investigating whether phytohormones including GAs impart any changes to the assembly and architecture of chloroplasts.

### 2.7. Jasmonates

Jasmonic acid (JA) and methyl jasmonate (MeJA), collectively referred to as jasmonates, are ubiquitously occurring lipid-derived compounds with various functions in plant growth and biotic and abiotic stress responses [171,172]. A recent study in citrus plants has demonstrated that transient accumulation of JA is essential for ABA increase, thus facilitating the survival of plants under severe drought conditions [173]. JA has also been reported to influence various photosynthetic parameters under high salt-stress conditions. The application of JA in rice plants exposed to high salinity mitigated the inhibitory effect of salt on the rate of ^14^CO_2_ fixation and showed improved *Fv*/*Fm*, leaf water potential, and a higher photosynthetic rate [69]. Similar results were reported by Velitcukova and Fedina [70] and Tsonev *et al.* [71] in *Pisum sativum* and barley plants, respectively, subjected to high salinity stress. More recently, a combination of noninvasive chlorophyll fluorescence imaging technology and RNA sequencing was used to determine the effect of JA on the growth, photosynthetic efficiency, and gene expression of *Arabidopsis* plants treated with coronatine, an agonist of JA receptors [174]. The reduced quantum efficiency of PSII, poor photosynthesis noted in coronatine-treated plants, suggested that these effects were tightly correlated with changes in the expression of genes involved in growth and photosynthesis [174].The application of MeJA at a specific concentration showed a protective effect against Cu and Cd ions and prevented the inhibitory effect of heavy metals on chlorophyll accumulation and photosynthetic activity, indicating that MeJA strongly influences heavy metal toxicity in *Arabidopsis* plants[72]. Similar changes were reported in the photosynthetic apparatus activity of JA-treated *Arabidopsis* plants under heavy metal stress, suggesting that JA, after the longest time, might enhance the sensitivity of *Arabidopsis* to Cu and Cd stress [73].Further studies are expected to improve our understanding of the effect of JAs on the relationship between photosynthesis and abiotic stress responses.

### 2.8. Salicylic Acid (SA)

Salicylic Acid (SA) is a well-known phenolic plant growth regulator that has gained a lot of attention chiefly because of its role in plants’ responses to biotic and abiotic stress [175,176]. Moreover, SA affects various aspects of plant growth and photosynthesis such as Rubisco activity, stomatal closure, chloroplast structure, and the accumulation of photosynthetic pigments [177] (Figure 1; Table 1). Controlled levels of SA in plants are considered an essential requirement for optimum photosynthesis and the maintenance of redox homeostasis [178]. Accumulating evidence indicates that SA plays a pivotal role in the protection of the photosynthetic apparatus of plants that are exposed to environmental stress [77,84,85,87,179]. SA affects photosynthesis via inducing stomatal closure and by slowing down PSII-ETC. However, the effects of SA application on PSII charge separation and stabilization were different in thylakoid samples and intact leaves, suggesting an indirect effect of SA on PSII [180]. Heat stress is particularly known to induce severe damage to the photosynthetic machinery by disrupting the OEC and the release of 33, 23, and 17 kDa proteins, and by the loss of cofactors, creating an imbalance in the photosynthetic ETC and causing damage to the D1 and D2 proteins [181,182,183]. The spraying of SA on grapevine leaves under heat stress reportedly induced thermotolerance by alleviating PSII damage, increasing photosynthetic capability, and adjusting the distribution of assimilates [83]. Later, a detailed study of SA pre-treatment on various components of PSII electron transport using OJIP analysis revealed that although SA treatment did not influence the photosynthesis under normal conditions, it significantly reduced the decline in *P*_N_ and the activation of Rubisco units in heat-stressed grapevine leaves [84]. The induction of thermotolerance with SA treatment in mustard and potato plants was associated with the observed decline in hydrogen peroxide (H_2_O_2_) content and catalase activity and it was proposed that both SA and H_2_O_2_ could be involved in signal transduction that facilitates the acclimation of plants under heat stress [184,185].

The influence of the foliar application of SA on carbon assimilation, Rubisco activity, and the photosynthetic carbon cycle were suggested as possible factors responsible for the induction of salt tolerance and the promotion of plant growth in wheat plants [75]. However, soaking barley seeds with SA prior to sowing also induced a pre-adaptive response to high salinity, which resulted in the protection of photosynthetic pigments and improved membrane integrity [186]. SA treatment through foliar spray in maize plants led to an improved *P*_N_ and increased the accumulation of photosynthetic pigments such as chlorophylls a and b and carotenoids [82]. A recent study on the influence of SA treatment in mustard plants exposed to salinity stress described that the application of 0.5 mM SA promotes photosynthesis and mitigates the effects of sodium chloride (NaCl)-induced salt stress via an increase in enzymes of the ascorbate-glutathione pathway. The application of SA displayed improvements in major photosynthetic and growth parameters such as *Fv*/*Fm*, *P*_N_, Rubisco activity, water use efficiency, stomatal conductance, intercellular CO_2_ concentration, leaf area, and plant dry mass, indicating the possible involvement of SA in the redox balance under high-salinity stress [179]. The application of 0.5 mM SA to *Torreya grandis* trees exposed to salt stress in a pot experiment was also reported to enhance the chlorophyll content, ROS enzyme activities, the *P*_N_, and mitigate membrane injury [87]. Foliar application of the same concentration (0.5 mM) of SA in *Vignaradiata* L. mitigated the NaCl-induced inhibition of photosynthesis through a decrease in Na^+^, Cl^−^, H_2_O_2_, thiobarbituric acid reactive substances, and electrolyte leakage, and increased the glutathione content coupled with the increased activity of ROS-scavenging enzymes [187]. Similarly, the low concentration (0.1 mM) of SA used in foliar treatment was reported to improve the PSII activity, the *P*_N_, the transpiration rate, and the activity of ROS-scavenging enzymes in cotton seedlings under NaCl-induced salt stress [81]. Apparently, the concentration of SA used to treat plants is a key factor in photosynthesis-related studies, as a high concentration of SA can also impose negative effects on the photosynthetic machinery. For example, the application of 10 mM SA to tobacco plants under non-stressed conditions had negative effects on stomatal conductance, CO_2_ assimilation, and ETC [180], while the application of 0.1 and 0.5 mM SA under stress conditions resulted in improved photosynthesis and plant growth [84,179]. However, contrary to these findings, a relatively higher concentration (5 mM) of SA was used for foliar application in Jatropha leaves exposed to salt stress [188]. It was reported that the SA efficiently mitigated the effects of salt stress by improving the *P*_N_ and enhancing the CO_2_ assimilation and the ROS-scavenging enzyme activities. Based on these findings, it is assumed that the effects of SA might depend on the dose as well as on the plant species. 

Beside conferring stress tolerance against salinity and heat stress, SA is also well documented for inducing tolerance against drought and metal toxicity through improved photosynthesis. Proteomic analysis of SA-treated wheat seedlings exposed to drought stress showed that 18 photosynthetic proteins were significantly upregulated, including luminal, oxygen-evolving enhancer, and PSII assembly factor proteins, providing clear evidence that SA stimulates the photosynthetic machinery when the plants face water deficits [77]. Furthermore, proteins involved in the antioxidant machinery such as ascorbate peroxidase, glutathione-S-transferase, 2-Cys peroxiredoxin, and dehydroascorbate reductase [77] indicate that SA treatment triggers the antioxidant machinery that mitigates drought-induced oxidative damage in plant cells. Numerous reports have documented that SA-treatment in plants subjected to drought exhibited a marked increase in their chlorophyll content, *Fv*/*Fm*, *P*_N_, stomatal conductance, and/or enhanced activities of ROS-scavenging enzymes in *Arabidopsis* [74], wheat [189,190,191,192,193,194], maize [187,195,196], rice [78,197], tomato [85], mustard [79,80], and zoysiagrass [198].

### 2.9. Strigolactones

Strigolactones (SLs) are a relatively new class of carotenoid-derived plant hormones known to regulate several developmental processes, particularly the regulation of shoot branching as well as the regulation of genes encoding *LHC* proteins and other photosynthetic units [33,199,200,201]. Few recent studies have indicated a possible involvement of SLs in plants’ responses to drought stress. Ectopic expression of the rice cystatin in *Glycine max* and *Arabidopsis* resulted in an increased accumulation of chlorophyll, enhanced shoot branching, and reduced mRNA abundance of SLs-related enzymes [202]. Interestingly, photosynthetic genes that are generally suppressed under drought conditions were found to be upregulated in *Arabidopsis* SL-signaling *max2* mutants in response to drought stress, indicating a putative involvement of SL-signaling in drought tolerance [88]. It must be noted that both SLs and ABA are carotenoid-derived hormones and SLs are known to induce *LHCB* genes, hence it is reasonable to predict a potential crosstalk between these two hormones and the light harvesting pathways. More efforts in the future could provide a better picture of these crosstalks and their relevance in the induction of abiotic stress tolerance in plants.

## 3. Future Challenges and Perspectives

As discussed earlier, abiotic stress-induced photoinhibition severely affects the physiology of plants mainly due to the inhibition of the PSII repair system. It is well-known that various events of the PSII repair system are associated with the redox state of the ETC [20]. Moreover, the role of various plant hormones and plant growth regulators in the expression of photosynthetic genes is also well studied [40,41,42,59,88]. Hence, it becomes imperative to focus on the influence of changes in hormonal signaling on photosynthetic events, including the PSII damage repair cycle. A recent study revealed some interesting facts about the involvement of CKs on highlight-induced photoinhibition and the expression of genes encoding proteases that play pivotal roles in the PSII damage repair cycle [39]. The expression of one of the three known C-terminus-processing proteases (AtCtpA1) showed a stronger upregulation under high-light stress in the control than in CK-deficient plants, suggesting that the high-light stress-responsive expression of that protease is regulated by CKs. Furthermore, analysis of AtCtpA1 mutants showed that these plants behave like control plants in terms of their response to high-light stress, indicating the putative involvement of some extra proteins in the regulation of the stress response [39]. Similarly, the involvement of other major phytohormones in the PSII damage repair cycle and its components cannot be ruled out. Seemingly, owing to the intricacies of hormonal crosstalk in plants, it is very challenging to decipher the precise roles of specific hormones in the fine-tuning of PSII damage repair and other photosynthetic events under stressful conditions. For example, although the antagonistic roles of CKs and ABA in regulating the stomatal function are well-known [203] (Figure 1), the crosstalk between these two hormones in regulating the expression of photosynthetic genes and the PSII damage repair cycle under stress-induced photoinhibition remains elusive. Similarly, ABA, GA, and ET interact with each other at various points during plant development as well as under abiotic stress [204,205] (Figure 1); however, it is not clear as to how these interactions influence the photosynthetic apparatus that facilitates the survival and acclimatization of plants under stress. The over-accumulation of ET in plants subjected to drought stress displayed enhanced senescence and disrupted ABA biosynthesis, resulting in reduced photosynthetic efficiency and slow leaf growth [4]. These observations indicated that a balance between ET and ABA levels might regulate plants’ responses to drought stress. These findings further indicate that extensive efforts are still required to gain a comprehensive understanding of interaction between these hormones. Plant osmolytes such as GB are synthesized in thechloroplasts and protect various luminal and stromal proteins, lipids, and enzymes [90]. Biosynthesis of GB is closely associated with the endogenous levels of ABA, SA, and ET and it has been suggested that excess electrons produced in the ETC during stress-induced photoinhibition can be consumed in the GB biosynthesis pathway, thereby inhibiting the over-reduction of the photosynthetic machinery [90]. Hence, it is speculated that a more comprehensive understanding of the interaction between stress-responsive hormones and plant osmolytes such as GB could greatly facilitate the development of photosynthetically efficient abiotic stress-tolerant crops.

Interestingly, the constitutive over-accumulation and exogenous application of hormones may exhibit entirely different effects on the photosynthetic machinery. For instance, the constitutive expression of the *ABA* gene showed negative effects on the biosynthesis of auxins and GAs that in turn hindered plant growth and development [206]. However, the exogenous application of ABA reduced the heat-induced PSII damage and induced thermostability in barley seedlings [43]. These findings suggest that plants producing more endogenous levels of ABA should be more tolerant to environmental stresses compared to control plants. This also reflects the large number of reports where hormonal metabolic pathways have been altered through genetic engineering to induce inherent abiotic stress tolerance in plants. At the same time, studies focusing on the influence of the exogenous application of hormones remain equally relevant; however, it is speculated that transcriptomics and proteomic analyses of plants’ responses to various hormones in mutants with altered photosynthesis could provide more details that would eventually facilitate crop-improvement programs. Furthermore, more efforts are warranted to elucidate the physiological relevance of altered expression reported in many abiotic stress-responsive genes, *LHCII* genes, and genes that encode intrinsic and extrinsic proteins of the PSII complex. In addition, metabolomic approaches are expected to play a significant role in understanding the influence of hormones on the metabolic responses of plants for the acclimation of the photosynthetic apparatus under environmental stress. A combination of datasets from different approaches would certainly enhance our knowledge of the physiological significance of phytohormones in photosynthetic efficiency under various environmental stress conditions.

## Abbreviations

ABA: abscisic acid
BR: brassinosteroid
CK: cytokinin
ET: ethylene
GA: gibberellic acid
IAA: indole acetic acid
IBA: indole butyric acid
LHCII: light harvesting complex II
NPQ: non-photochemical quenching
OEC: oxygen-evolving complex
OJIP: O and P denote origin and peak respectively and J-I denote intermediate phases of fluorescence induction
*P*_N_: net photosynthesis rate
PsbO: photosystem b *O* protein
PsbP: photosystem b *P* protein
PSI: photosystem I
PSII: photosystem II
ROS: Reactive oxygen species
SA: salicylic acid
VDE: violaxanthin de-epoxidase
